# Alternative Approach to Left Ventricular Thrombectomy Using Percutaneous Cardiopulmonary Bypass: A Case Report

**DOI:** 10.7759/cureus.77229

**Published:** 2025-01-10

**Authors:** Rishabh Kasarla, Maria Beyer, Sebastion Cuello, Pranav Tadepalli, Erik Beyer

**Affiliations:** 1 Cardiothoracic Surgery, Nova Southeastern University Dr. Kiran C. Patel College of Allopathic Medicine, Davie, USA; 2 Cardiothoracic Surgery, University of Texas Health Science Center at San Antonio, San Antonio, USA; 3 Cardiothoracic Surgery, American University of the Caribbean School of Medicine, Cupecoy, SXM; 4 Cardiothoracic Surgery, Nova Southeastern University College of Osteopathic Medicine, Davie, USA; 5 Cardiothoracic Surgery, Palmetto General Hospital, Hialeah, USA

**Keywords:** cardiac surgery innovation, cardiopulmonary support techniques, high-risk embolization, left ventricular thrombus, modified dor procedure, no-touch surgical approach, percutaneous cardiopulmonary bypass, stroke prevention, thrombectomy, ventriculotomy

## Abstract

Traditional bypass methods for intracardiac thrombectomy may require direct manipulation of the heart, which carries the potential for increased stroke risk from either arrhythmias or direct dislodgement and embolization of a thrombus. Currently, there is very little research regarding the different ways to approach cardiopulmonary bypass in thrombectomy cases. This case study highlights the use of percutaneous femoral access cardiopulmonary bypass (PCPB) in the management of a large, mobile, left ventricular thrombus (LVT) in a patient with a recent stroke. In this case, a 49-year-old male with a history of an anterior wall myocardial infarction and subsequent percutaneous coronary intervention (PCI) presents now, four years later, with shortness of breath. At the time of his initial presentation, he was documented to have anterior wall akinesis and a mobile LVT. Warfarin was recommended at that time, but the patient was noncompliant. Before further workup could be completed, the patient experienced a cerebrovascular accident (CVA), but after a comprehensive workup, he was cleared for surgery two days later. The patient required surgery for apical thrombus removal and ventricular reconstruction. Following sternotomy and initiation of percutaneous cardiopulmonary bypass, an apical infarct and thrombus were identified and removed via left ventriculotomy. Purse-string sutures were placed to exclude scar tissue and restore ventricular shape, and the ventriculotomy was closed using a modified Dor procedure. The patient was successfully weaned off bypass. The patient was discharged on postoperative day 12 with normalized labs, showed excellent functional recovery, remained symptom-free at two months, and remained stable without complications at eight months. The "no touch" approach provided by percutaneous bypass minimizes the risk of complications such as a stroke, suggesting its consideration as a standard of care during left ventricular thrombectomy. This case encourages further research into PCPB as a preferable alternative to traditional bypass methods in managing left ventricular thrombi, especially in high-risk embolization scenarios.

## Introduction

Left ventricular thrombus: etiology and clinical presentation

Left ventricular thrombus (LVT) is a significant complication following anterior ST-segment elevation myocardial infarction (STEMI), with an overall incidence of approximately 10% [1]. Among STEMI patients undergoing percutaneous coronary intervention (PCI), the incidence is slightly lower, around 4% [1]. LVT develops as a result of Virchow’s triad: endothelial injury, blood stasis due to left ventricular dysfunction, and hypercoagulability triggered by systemic inflammation [2]. These pathological changes, often initiated by myocardial ischemia and infarction, set the stage for thrombus formation.

The clinical presentation of LVT varies depending on its acuity. Acute LVT often manifests with sudden, severe symptoms and is strongly associated with an elevated risk of systemic embolism, stroke, major cardiovascular events, and mortality. In contrast, chronic LVT tends to have a more insidious course, with gradually worsening symptoms that may go unnoticed until significant complications arise [3]. Thrombus size and morphology also differ widely between patients. In one study that assessed a group of 77 patients, the average width of the thrombus within the left ventricle was between 10 mm and 12 mm [4].

Treatment

The management of LVT involves a combination of medical and surgical strategies tailored to the risk of embolization and individual patient characteristics. The most common means of treating LVT include direct oral anticoagulants, vitamin K antagonists, antiplatelet therapy, and surgical thrombectomy [3]. The choice of therapy depends on predictors of embolism such as thrombus protrusion into the left ventricular cavity, thrombus mobility, thrombus size, patient age over 68, and recurrence of LVT. Immobile thrombi are likely to be chronic and less likely to embolize. For these instances, the preferred treatment is non-invasive, such as anticoagulant therapy [3]. In situations with a high risk of embolization, such as higher mobility or size, surgery is favored. The surgical approach to left ventricular masses can be through the left atrium, aorta, or left ventricle [5]. In our case, a left ventriculotomy was performed to remove the thrombi, selected based on its specific size and location.

Surgical approaches to LVT removal depend on factors such as thrombus size, location, and patient comorbidities. Traditional cardiopulmonary bypass (TCPB) is the standard approach to open-heart surgical technique and allows complete control over the surgical field. It involves cannulation of the right atrium before the induction of cardioplegia. Percutaneous cardiopulmonary bypass allows access through the femoral artery and vein, without direct cannulation of the heart. One advantage of percutaneous femoral access cardiopulmonary bypass (PCPB) is its utility for prophylactic support in patients who develop complications such as cardiac arrest or cardiogenic shock during high-risk catheterization procedures, as the initiation of PCPB can be completed rapidly, typically within about 20 minutes [6,7]. PCPB has been applied in various critical scenarios, including refractory cardiac arrest from acute myocardial infarction (MI) or failed angioplasty, massive pulmonary emboli, high-risk angioplasty, hypothermia, and refractory arrhythmia [8]. It cannot be used if the patient has iliofemoral disease and not for more than 24 hours, due to the disruption of blood components [6].

## Case presentation

A 49-year-old male with a complex medical history presented to the hospital experiencing shortness of breath. His medical history was notable for coronary artery disease (CAD), atrial fibrillation (Afib), hypercholesterolemia, and MI in 2019, where he underwent a PCI of the proximal left anterior descending (LAD) artery. At that time, he was diagnosed with anterior wall akinesis and an associated apical thrombus. Despite recommendations for warfarin therapy to manage his condition, he was noncompliant. The patient’s non-compliance with warfarin therapy may have been influenced by factors such as poor medication adherence and insufficient follow-up, which are often observed challenges in long-term anticoagulation management.

During his current admission, cardiac catheterization confirmed the patency of the previously placed stent in the proximal LAD and showed no abnormalities in the circumflex and right coronary arteries (RCA). While in the hospital, the patient experienced a cerebrovascular accident (CVA). After a comprehensive neurology workup, he was cleared for surgery after two days.

Surgical intervention

A median sternotomy was performed (Video 1), the pericardium was opened in the midline, and the heart was inspected. Femoral cardiopulmonary bypass was initiated after ultrasonographic guidance was used to place cannulae in the femoral artery and vein. Once adequate heparinization was confirmed, the bypass was initiated, and the aorta was cross-clamped. Antegrade cardioplegia was then delivered via the aortic root.

**Video 1:** Surgeon performing left ventricular thrombectomy using percutaneous cardiopulmonary bypass

**Figure 1:**
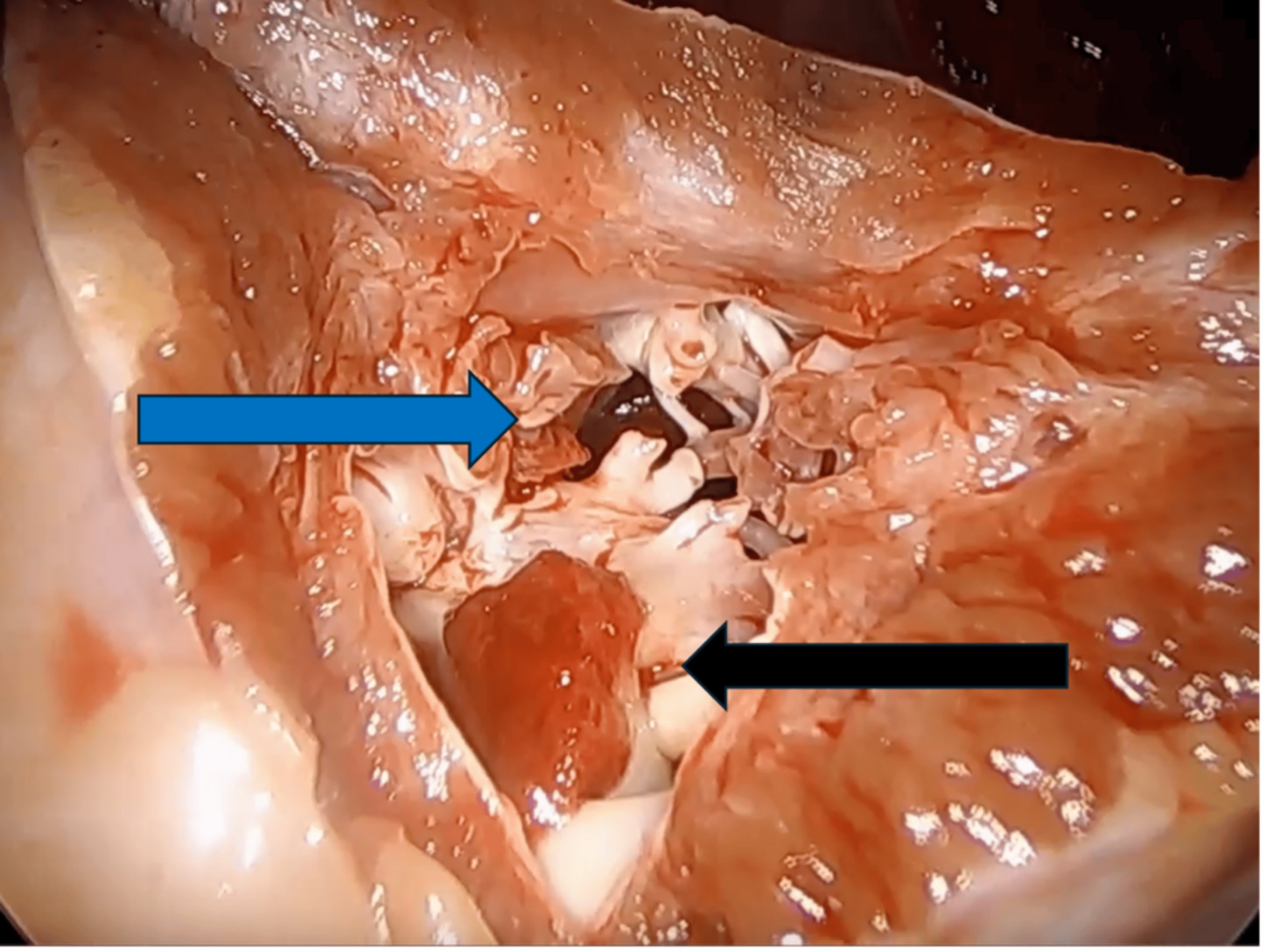
The image shows a small superficial thrombus indicated by the black arrow and a larger, deeper thrombus marked by the blue arrow within the trabeculated scar tissue of the left ventricle

Ventriculotomy at the apex of the left ventricle revealed scarred, trabeculated tissue from the previous MI. This scar tissue was resected around the thrombus site to ensure clear margins and provide access into the ventricle. Then the thrombus itself was dissected and removed (Figure 1). During this process, meticulous care was taken to ensure the complete removal of all thrombotic material, eliminating any residual particles that might cause further embolization.

Following the removal of the thrombus, the transition zone between the scarred myocardium and the healthy tissue was identified. Ventricular restoration was accomplished using a modified Dor procedure [9]. Two purse-string sutures were placed circumferentially at the base of the scar adjacent to the normal myocardium, excluding the akinetic segment of the ventricular apex. The ventriculotomy was then closed in two layers using two large strips of felt as buttresses to facilitate hemostasis. The aortic cross-clamp was removed, and warm blood cardioplegia was administered. The heart resumed a spontaneous rhythm, the patient was weaned from cardiopulmonary bypass and decannulated, protamine was administered to reverse anticoagulation, and hemostasis was achieved.

Results

Intraoperative Findings

**Figure 2:**
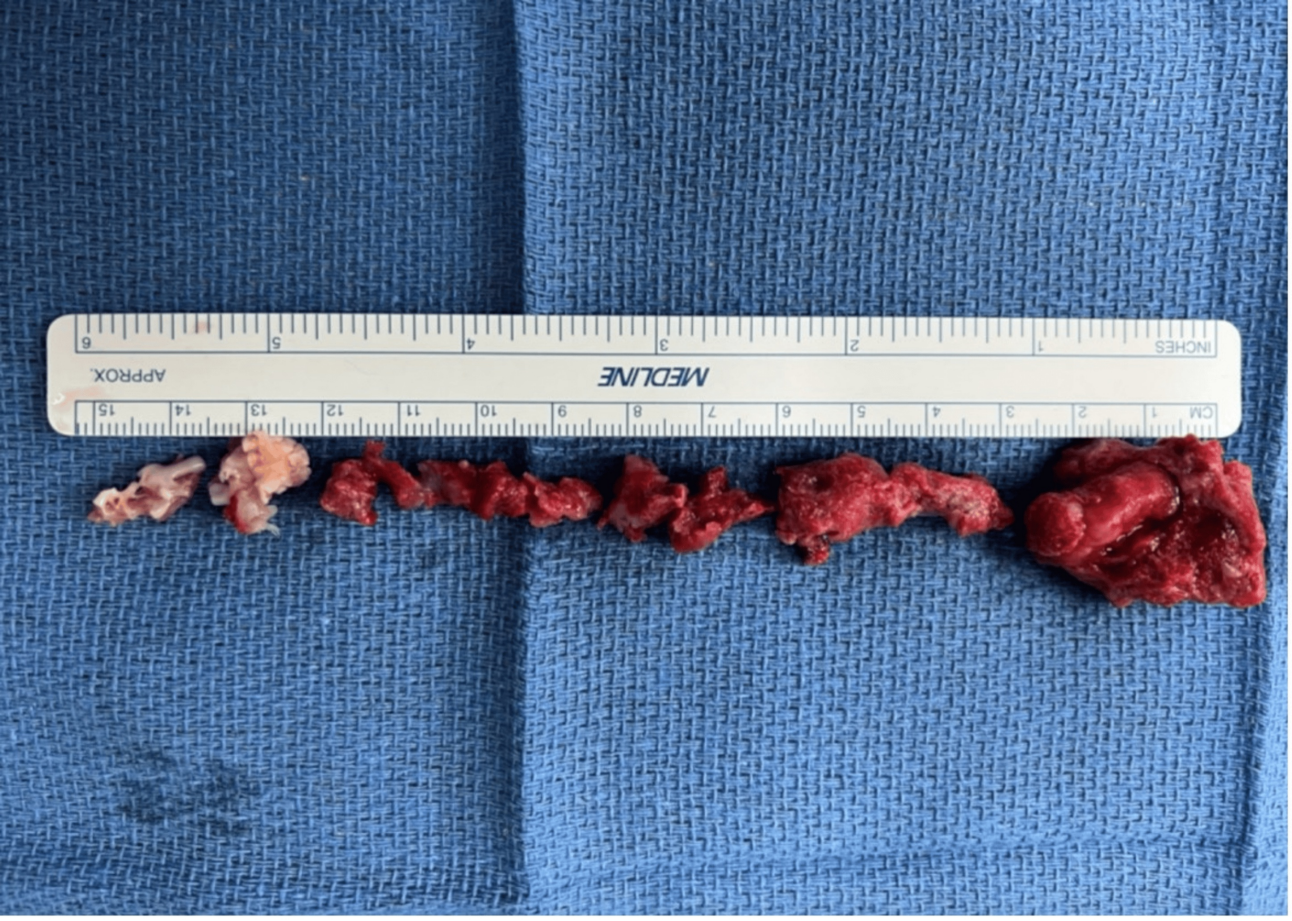
Specimens of thrombi successfully removed from the left ventricle during the thrombectomy

The operation revealed large thrombi within the left ventricle, which were removed without complication (Figure 2).

Follow-Up

At his two-month follow-up, the patient exhibited excellent functional status, with significant improvements in symptoms of heart failure, including a marked enhancement in functional capacity, absence of dyspnea, and stable vital signs, with no evidence of ascites. Eight months post-surgery, he maintains a stable condition with no cardiovascular complications.

## Discussion

Our patient presented with shortness of breath, four years after an anterior MI, who had been noncompliant with anticoagulation therapy for an LVT. He was found to have multiple large thrombi that were treated with a left ventricular thrombectomy with percutaneous cardiopulmonary bypass. The successful use of percutaneous cardiopulmonary bypass in our case highlights its potential utility in managing left ventricular thrombi.

Ventricular thrombectomy is usually performed using TCPB, which often involves cannulation of the right atrium before aortic cross-clamp and cardioplegia are induced. We emphasize that traditional bypass often involves manipulation of the heart, increasing the risk of embolization. Specifically, cannulation of the right side of the heart can provoke arrhythmias or exert direct pressure, potentially disturbing and dislodging the thrombus, leading to embolization.

This case demonstrates the utility of percutaneous cardiopulmonary bypass (PCPB) as an effective alternative to traditional bypass for the management of an LVT. PCPB reduces direct cardiac manipulation and the risk of embolization, making it especially advantageous in high-risk cases where traditional bypass methods might increase the chances of thrombus dislodgment and systemic embolization. Its application may also extend to other scenarios involving a high risk of distal embolization from intra-cardiac masses or objects, such as dislodged septal defect closure devices, transcatheter aortic valve replacement (TAVR)-related complications, clot-in-transit from deep vein thrombosis, guidewire malplacements, or mobile intra-cardiac tumors.

While PCPB streamlines certain procedural aspects, it also presents technical challenges. These include challenges related to vascular access, risks associated with arterial cannulation, and potential complications involving blood flow and circulation management. Though PCPB may increase procedural costs in some cases due to specialized equipment and the need for expertise, it has the potential to offset these costs by reducing complications and improving patient outcomes. Given its advantages in managing complex cases, further exploration of PCPB is warranted to optimize its application and establish its role as a potentially effective alternative in modern cardiac surgery.

## Conclusions

Embolization is a feared complication of ventricular thrombi, which are typically treated with anticoagulation therapy. In cases requiring surgical intervention, the "no-touch" approach of percutaneous cardiopulmonary bypass minimizes the risk of embolic complications, such as stroke. Future research comparing traditional bypass approaches to percutaneous bypass, including randomized controlled trials and observational studies, is essential to address the need for further research into their effects on mortality, morbidity, and cost in this patient population. Leveraging multi-center collaborations and large patient registries will be crucial for advancing the evidence base and optimizing the clinical application of PCPB.
